# LONGITUDINAL ASSOCIATION OF COGNITIVE IMPAIRMENTS WITH LATE-LIFE WEALTH CHANGES AMONG OLDER AMERICANS

**DOI:** 10.1093/geroni/igad104.3080

**Published:** 2023-12-21

**Authors:** Darlingtina Esiaka, Ashly Westrick, Ronica Rooks, Helen Meier, Mark Manning, Wassim Tarraf

**Affiliations:** University of Kentucky, Lexington, Kentucky, United States; University of Michigan School of Public Health, Ann Arbor, Michigan, United States; University of Colorado Denver, Denver, Colorado, United States; University of Michigan, Ann Arbor, Michigan, United States; Oakland University, Rochester, Michigan, United States; Wayne State University, Detroit, Michigan, United States

## Abstract

Disparities in wealth in the US are linked to disadvantageous health outcomes over the lifecourse. Cognitive impairment can widen wealth disparities. We examine how cognitive impairment influences change in wealth starting in mid-life and into older adulthood and the role of social determinants of health in wealth loss due to cognitive impairment. We use biennial longitudinal data (Health and Retirement Study: 2000-2018) on Non-Hispanic White (NH-W), NH-Black (NH-B), and Hispanic (H) middle-aged adults (51-64 years and cognitively healthy in 2000; unweighted n=3,651) to model wealth change (measured as the relative (%) difference in reported wealth between 2000-2018 [in 2018 dollars]) as a function of cognitive status (using three latent phenotypes derived from longitudinal latent class analyses: cognitively healthy [CH; 77%], increasing cognitive impairment [ICI; 19%], and increasing dementia [ID; 4%]). We fit racial/ethnic stratified regression models to examine relationships between cognitive phenotypes and wealth change, adjusting for covariates. Using Blinder-Oaxaca decomposition techniques, we examined the contributions of predisposing (e.g. race/ethnicity), health enabling (e.g. insurance source), and health needs (i.e. chronic conditions) factors in wealth change by cognitive phenotypes. Results show NH-Ws had higher levels of reported wealth in both 2000 and 2018, compared to NH-Bs and Hs. However, the relative rate of change in wealth was not-significantly different across race/ethnic groups. Education, insurance, and health explained most differences in wealth change related to cognitive impairment. Yet, nearly 50% of group differences in wealth changes remained unexplained. Future work should further elucidate sources of these unexplained differences, overall and by race/ethnicity.

